# A survey about label enhancement methods for parenteral medication in European hospital pharmacies

**DOI:** 10.1007/s00228-020-02916-x

**Published:** 2020-06-20

**Authors:** K. H. M. Larmené-Beld, R. N. Keers, K. Taxis

**Affiliations:** 1 Department of Clinical Pharmacy, Isala Hospital, Dokter van Heesweg 2, 8025 AB Zwolle, The Netherlands; 2 Unit of Pharmacotherapy, Epidemiology & Pharmacoeconomics (PTE2), Groningen Research Institute of Pharmacy, University of Groningen, Groningen, The Netherlands; 3 Centre for Pharmacoepidemiology and Drug Safety, Division of Pharmacy and Optometry, School of Health Sciences, The University of Manchester, Manchester, UK; 4 Pharmacy Department, Greater Manchester Mental Health NHS Foundation Trust, Manchester, UK

**Keywords:** Look-alike, Label enhancement, Parenteral medication, Hospital

## Abstract

**Purpose:**

Unclear labeling has been recognized as an important cause of look-alike medication errors. Little is known about which labeling practices are currently used in European hospitals. The aim of this article is to obtain an overview of the labeling practices for parenteral medications, in relation to national guidelines, in the Netherlands, Germany, and the UK.

**Methods:**

An online survey was conducted using the Qualtrics® software. The survey was distributed to hospital pharmacists in the Netherlands, Germany, and the UK. The results were downloaded from Qualtrics and exported to Microsoft Excel. Data were categorized into groups and analyzed descriptively.

**Results:**

In total, 104 responses were received. The response rate was 63% (*n* = 48) in the Netherlands and 11% (*n* = 41) for Germany; for the UK, 15 responses were received. In general almost 90% of the respondents followed the National guidelines concerning labeling of pharmacy-prepared parenteral products. The use of label enhancement techniques was relatively low in all countries. On average, the use of “Tall Man” lettering was 19%, the use of color coding was 29%, and the use of a barcode on the label was 27%.

**Conclusion:**

Label-enhancement methods for parenteral medication in hospital pharmacies do not seem to be widely implemented and acknowledged in European hospitals, but response rates were limited for two countries. Greater standardization in conjunction with research for evidence-based enhancement techniques is needed to guide improvement in labeling practices across Europe.

## Introduction

Hospital pharmacists have an important role in promoting and ensuring the safe use of medicines [1]. Good labeling of medication is recognized as an important aspect of medication safety in hospitals, but 20% or more of medication errors may be related to confusing packaging and poor labeling [2, 3]. The basic function of a medication label is to enable correct identification and safe administration of the product. So-called look-alike labels, because of similar drug names (e.g., dobutamine–dopamine) or otherwise similar appearance of the labels, could result in the administration of the wrong drug. In particular for parenteral medications, this can have serious consequences for patients [4–6]. Many parenteral medications involve high-risk medication with a narrow therapeutic range. Few studies have focused on labeling issues in parenteral medication. In anesthesia, Abeysekera et al. have shown that 20% of reported medication errors involved labeling errors of syringes [7].

Enhancing the readability of labels could be done in different ways [4, 5]. Closed loop systems using barcode technologies have been introduced in some hospitals [1, 8]. However, medication labels need to still be easily readable for humans, for example in emergency situations. “Tall Man” lettering is regarded in the literature as a potential solution; this approach aims to maximize the difference between two similar drug names and avoid confusion by capitalizing part of the drug names [9]. A recently published systematic review found evidence from laboratory-based studies that “Tall Man” lettering contributes to reduced error rates, which the authors suggested was due to a better readability of medication labels, but evaluations in real-life settings are needed to strengthen this conclusion [10]. Several national health organizations have endorsed “Tall Man” lettering including the Joint Commission International (JCI) and the Institute for Safe Medication Practices (ISMP) in the USA [9, 11, 12]. The EMA guideline incorporated the use of capital letters, but did not explicitly mention Tall Man lettering and its purposed use for distinction between words [6]. Elsewhere in anesthesia, color-coded labels are used to distinguish between different substance classes. This approach is described in an international standard (ISO 26825) [13], but a recent systematic review reported that there is little published evidence supporting enhanced readability by using color-coding [10].

Internationally, there is no consensus about the content and form of parenteral medication labels. There is also a lack of guidance from the FDA and European Medicines Agency (EMA) [6, 11]. This concerns the labels produced by manufacturers as well as labels produced in the hospitals by the hospital pharmacies or on the wards. As little is known about which labeling practices are currently used in European hospitals, a survey across countries may therefore be useful to identify best practices and drive improvement [1].

Therefore, we conducted a survey among hospital pharmacists to obtain an overview of the labeling practices for parenteral medications, in relation to national guidelines, in the Netherlands, Germany, and the UK.

## Methods

An online survey using the Qualtrics® software was developed. The survey was divided into three parts; the first part concerned the general information of the hospital pharmacy (type, place, number of beds, etc.), the second asked participants to describe label-enhancement methods used for parenteral medication prepared by their hospital pharmacy, and the final part consisted of questions about their hospitals’ labeling policy for parenteral medication prepared and used on hospital wards. The survey was distributed in the Netherlands, Germany, and the UK. In the Netherlands, the survey was sent to all heads of hospital pharmacies using a list of contact details obtained from the Dutch Association of Hospital Pharmacists. In Germany, the survey was distributed to all hospital pharmacists based on the contact information of 392 German hospital pharmacies obtained through the Bundesverband Deutscher Krankenhausapotheker (ADKA). The UK survey was distributed by a contact at the UK Pharmaceutical Aseptics Services Group (PASG) to the members of the North West Aseptic Services Group (ASG) and the wider PASG. The survey for the Netherlands was launched in January 2016, for the UK and Germany in June 2017. The questions in the survey included a mix of multiple choice and open questions. After 2 weeks, a reminder was sent by email; a second reminder (also by email) was sent another week later. The results were downloaded from Qualtrics and exported to Microsoft Excel. Data were categorized into groups and analyzed descriptively. Discrepancies or unclear answers were verified with the respondent (*n* = 2).

## Results

Overall, 104 responses were received where each response represented an individual hospital. The response rate was 63% (*n* = 48) in the Netherlands and 11% (*n* = 41) for Germany. A response rate could not be calculated for the UK (15 respondents) as it was unknown how many hospital pharmacists received the survey. From the respondents, respectively, 31, 36, and 15 hospitals have a production facility in the pharmacy and were taken into further analysis. Most respondents reported that their hospital pharmacies followed relevant national guidelines concerning labeling of pharmacy-prepared parenteral products, with 84, 92, and 95% in agreement respectively for the Netherlands, Germany, and the UK. The use of label-enhancement methods used by pharmacies varied between the countries. The use of “Tall Man” lettering was relatively low in all countries, respectively five (5/ 31, 16.1%), five (5/ 36, 13.9%), and four (4/15, 26.4%) for parenteral medications in the Netherlands, Germany, and the UK.

In Germany, half (50%) of the respondents used color coding, while only 16% and 20% of the respondents used this strategy respectively in the Netherlands and the UK. Of the pharmacies in Germany using color coding, six (6/18, 33.3%) used color coding according to the ISO 26825, two (2/18, 11.1%) used the ISO 26825 partially but combined this with their own color coding, and ten (10/18 55.6%) did not follow the ISO 26825 but used their own coloring system to differentiate between specific drugs. Some pharmacies used a combination of color with another enhancement technique, e.g., in Germany, one pharmacy (1/18) used color coding in combination with a barcode, and four pharmacies (4/18) used color in combination with “Tall Man” lettering.

A total of 55% (*n* = 17/31) of the respondents with a production facility in the Netherlands used a barcode or data matrix code on the label and these were 6% and 20% in Germany and the UK (see Table 1 for all results).

**Table 1:** Results survey

General information	The Netherlands (n = 76)		Germany (*n* = 392)		The UK (*n* = 15)	
	*n*	%	*n*	%	*n*	%
Overall response rate	48	63.2	41	10.5	15	#
Hospital type						
Local	28	58.3	7	17.1	8	53.3
Tertiary hospital	16	33.3	11	26.8	7	46.7
Academic	4	8.3	17	41.5	0	
Other			3	7.3		
Production facility pharmacy	31	64.6	36	87.8	15	100.0
Applying national guideline for labeling*	26	83.9	33	91.7	14	93.3
Using color coding	5	16.1	18	50.0	3	20.0
Using Tall Man lettering	5	16.1	5	13.9	4	26.7
Using pictograms/icons	2	6.5	0	0	0	0
Using barcoding/data matrix code	17	54.8	2	5.6	3	20.0
Warning signs epidural medication	4	12.9	24	66.7	10	66.7
Warning sign multiple doses	11	35.5	15	41.7	4	26.7

#No percentage could be calculated because it was unknown how many pharmacists received the survey

*National labeling guidelines: the Netherlands: guideline labeling Dutch Association of Hospital Pharmacists (2011); Germany: Apothekenbetriebsordnung (ApBetrO § 14); the UK: “*Best practice guidance on labelling and packaging of medicines*” from the MHRA and “*Medicines, Ethics and Practice - The Professional Guide for Pharmacists*” from the Royal Pharmaceutical Society

## Discussion

**Fig. 1:**
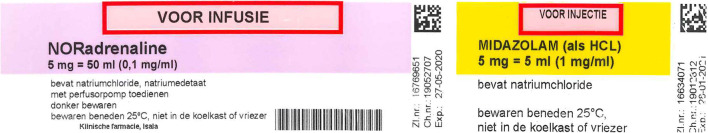
Examples of labels using Tall Man lettering, data matrix, and barcoding

**Fig. 2:**
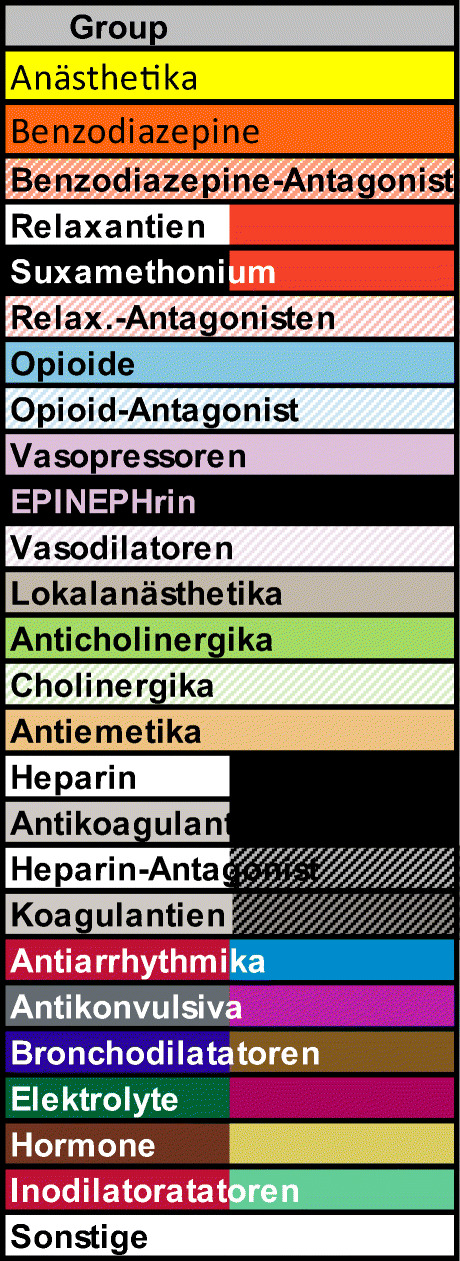
Overview of the color-coding scheme developed by the German Interdisciplinary Association for Intensive Care and Emergency Medicine (Deutsche Interdisziplinäre Vereinigung für Intensiv- und Notfallmedizin, DIVI) [15]

This is the first study to explore the labeling practices for parenteral medications in European hospital, and the results provide important insight concerning the use of methods of label enhancement of parenteral medications in hospital pharmacies. Label-enhancement methods were not widely used in the Netherlands, Germany, or the UK, with differences between the countries in approaches they did employ. While in Germany, color coding was the most commonly used method, in the Netherlands, this was the barcode/data matrix code method. Figure 1 shows examples of labels using Tall Man lettering, barcode, and data matrix beside the ISO26825; in Germany, they also used the DIVI system; a color-coding system developed by the Deutsche Interdisziplinäre Vereinigung für Intensiv-und Notfallmedizin for labeling on the ward. Figure 2 shows an overview of the DIVI color scheme [14, 15]. In the guideline of the UK, color is mentioned as an option to enhance labels, but no reference to the ISO 26825 guidance is included [16]. In the Dutch labeling guideline, no conclusive advice is provided about the use of color on labels [17]. Every hospital can decide whether or not to use color on labels, and those that do are free to use their own color system instead of the international ISO color-coding system. This is because of a number of limitations including the existence of more look-alike drugs or drug groups than there are colors that could be used in the ISO color-coding system, and the prevalence of congenital color vision deficiency which affects about 8% of men and 0.4% of women in the general population [18]. Despite these limitations, more importantly, evidence suggests that healthcare professionals will rely solely on the color of the labels, and not read the labels at all [19, 20]. Remarkably, many hospital pharmacists in Germany and the UK were not aware (based on the comments as free text in the survey) of the possibility of using “Tall Man” lettering and pictograms as a label-enhancement method.

One of the main limitations of this survey is the low response rate of the survey in Germany and the UK. Despite the reminder emails that were sent, the response rate remained low.

Our findings give valuable insights into the labeling practices of hospital pharmacy production units in the Netherlands, Germany, and the UK and this is the basis for further work. Our results suggest that a first step is creating more awareness among European hospital pharmacists about the different methods of label enhancements. In light of the variety of approaches utilized and lack of specificity in national guidelines, more research is urgently needed to strengthen the evidence base on safe labeling methods in order to guide practice [10]. Such research needs to be approached from a practice-perspective, as labeling is only one aspect of a multifaceted approach to improve safe medication use in hospitals including procedures such as double checking [21, 22] or safe preparation of aseptic products [23]. Also the use of computerized decision support in combination with a barcode on the label may further decrease the risk of medication errors. In a survey in 2017 of the European Associations of Hospital Pharmacists, 45% of the respondents reported use of computerized decision support, with high variation between countries. For example, 90% of the respondents in the Netherlands used decision support, whereas less than 40% in the UK did so [1]. Multicenter studies are needed to evaluate the impact of interventions in practice settings as shown in a recent study completed in the USA, evaluating the impact of introducing “Tall Man” lettering on prescription errors [24]. Collaborations across European countries could be very useful to drive this agenda forward. Eventually, standardization across Europe should improve safety, as using different color-coding systems, could create confusion and compromise patient safety. A European guideline summarizing current evidence and best practices could be a first step in achieving this goal.

## Conclusion

Our study in the Netherlands, Germany, and the UK suggests that label-enhancement methods for parenteral medication in hospital pharmacies are not widely implemented and acknowledged in hospitals in these countries. Hospitals use national guidelines but also various locally developed methods for label enhancement. A larger-scale survey with a better response rate would be needed to confirm if this a European-wide issue. Greater standardization in conjunction with research for evidence-based enhancement techniques is needed to guide improvement in labeling practices.

## References

[1] Horák P, Underhill J, Batista A, Amann S, Gibbons N (2018). EAHP European Statements Survey 2017, focusing on sections 2 (Selection, Procurement and Distribution), 5 (Patient Safety and Quality Assurance) and 6 (Education and Research). Eur J Hosp Pharm.

[2] Thomas MR, Holquist C, Phillips J (2001). Medication error reports to FDA show a mixed bag. FDA Saf Page.

[3] Berman A (2004). Reducing medication errors through naming, labeling, and packaging. J Med Syst.

[4] Berdot S, Roudot M, Schramm C, Katsahian S, Durieux P, Sabatier B (2016). Interventions to reduce nurses’ medication administration errors in inpatient settings: a systematic review and meta-analysis. Int J Nurs Stud.

[5] Keers RN, Williams SD, Cooke J, Ashcroft DM (2013). Causes of medication administration errors in hospitals: a systematic review of quantitative and qualitative evidence. Drug Saf.

[6] European Commission (2009) Guideline on the readability of the labelling and package leaflet of medicinal products for human use. Revision 1

[7] Abeysekera A, Bergman IJ, Kluger MT, Short TG (2005). Drug error in anaesthetic practice: a review of 896 reports from the Australian Incident Monitoring Study database. Anaesthesia.

[8] McLeod M, Ahmed Z, Barber N, Franklin BD (2014). A national survey of inpatient medication systems in English NHS hospitals. BMC Health Services Research.

[9] Institute for Safe Medication Practices. FDA and ISMP lists of look-alike drug names with recommended Tall Man letters. Available at: http://www.ismp.org/tools/tallmanletters.pdf. Accessed 21 Jun, 2016.

[10] Larmene-Beld KHM, Alting EK, Taxis K (2018) A systematic literature review on strategies to avoid look-alike errors of labels. Eur J Clin Pharmacol 74(8):985–993 10.1007/s00228-018-2471-z PMC6061459 29754215

[11] U.S. Department of Health and Human Services, Food and Drug Administration, Center for Drug Evaluation and Research (CDER) (2013) Guidance for industry; safety considerations for container labels and carton labeling design to minimize medication errors

[12] Joint Commission International (2013). Joint Commission International Accreditation Standards for hospitals.

[13] ISO International Standards. ISO 26825 (2008) Anaesthetic and respiratory equipment: user-applied labels for syringes containing drugs used during anaesthesia; colours, design and performance. ISO International Standards 2008

[14] Sybrecht GW (2010). Empfehlung zur Kennzeichnung von Spritzen in der Intensiv-und Notfallmedizin. Anasth Intensivmed.

[15] Deutsche Interdisziplinäre Vereinigung für Intensiv- und Notfallmedizin. DIVI-Standard-Spritzenetiketten: Gruppenzuordnung. Available at: https://www.divi.de/empfehlungen/qualitaetssicherung-intensivmedizin/spritzenetiketten. Accessed 29 october, 2019.

[16] Royal Pharmaceutical Society of Great Britain (2015) Medicines, ethics and practice 39: the professional guide for pharmacists. Pharmaceutical Press

[17] Werkgroep etikettering Dutch Association of Hospital pharmacists (2011) Richtlijn etikettering van apotheekbereidingen. pp 1–56

[18] Spalding JA (1999). Colour vision deficiency in the medical profession. Br J Gen Pract.

[19] Filiatrault P (2009). Does colour-coded labelling reduce the risk of medication errors?. Can J Hosp Pharm.

[20] Institute for Safe Medication Practices (2003) How color-coding products, such as cyclopentolate hydrochloride 1% solution and tropicamide 1% solution, can lead to medication errors. Acute care ISMP Medication Safety Alert

[21] Schwappach DL, Pfeiffer Y, Taxis K (2016) Medication double-checking procedures in clinical practice: a cross-sectional survey of oncology nurses’ experiences. BMJ Open 6(6):e01139–e011394 10.1136/bmjopen-2016-011394 PMC4916573 27297014

[22] Schwappach DLB, Taxis K, Pfeiffer Y (2018). Oncology nurses’ beliefs and attitudes towards the double-check of chemotherapy medications: a cross-sectional survey study. BMC Health Serv Res.

[23] Larmené-Beld K, Frijlink H, Taxis K (2019). A systematic review and meta-analysis of microbial contamination of parenteral medication prepared in a clinical versus pharmacy environment. Eur J Clin Pharmacol.

[24] Zhong W, Feinstein JA, Patel NS, Dai D, Feudtner C (2016). Tall Man lettering and potential prescription errors: a time series analysis of 42 children’s hospitals in the USA over 9 years. BMJ Qual Saf.

